# Pazopanib in patients with primary multi-metastatic bone Ewing sarcoma

**DOI:** 10.3389/fonc.2025.1653015

**Published:** 2025-10-23

**Authors:** Anna Raciborska, Katarzyna Bilska, Jadwiga Węcławek-Tompol, Dorota Sega-Pondel, Anna Zelwiańska, Borys Przybyszewski, Radosław Chaber, Renata Tomaszewska, Justyna Antoniuk-Majchrzak, Tomasz Koziński

**Affiliations:** ^1^ Department of Oncology and Surgical Oncology for Children and Youth, Institute of Mother and Child, Warsaw, Poland; ^2^ Department and Clinic of Pediatric Oncology, Hematology and Bone Marrow Transplantation, Wroclaw Medical University, Wroclaw, Poland; ^3^ Department of Pediatric Oncohematology, Rzeszow University, Rzerzow, Poland; ^4^ Department of Pediatric Hematology and Oncology, Silesian Medical University, Zabrze, Poland

**Keywords:** pazopanib, metastatic Ewing sarcoma, children, treatment, multi kinase inhibitors

## Abstract

**Background:**

Despite the use of different treatment regimens, patients with primary multi-metastatic Ewing sarcoma disease have a dismal outcome. Lately, pazopanib has been proposed as an effective salvage regimen for soft tissue sarcoma (STS), including extraosseous Ewing sarcoma (ESS). Thus, we sought to evaluate this approach for young patients with primary multi-metastatic bone Ewing sarcoma.

**Materials and methods:**

Eleven patients with primary multi-metastatic bone Ewing sarcoma (metastasis to the bone and/or bone marrow), received standard first-line treatment in parallel with pazopanib. All patients had standard tumor imaging and laboratory evaluation. All toxicities were documented.

**Results:**

Pazopanib was administered throughout the whole treatment period (paused during the surgical procedure) and after its completion, on average 1.7 years (range 0.9 to 5.1). At the time of the beginning of pazopanib, the median age was 14.2 years (range 5.1 to 17.8 years). The primary tumor was operated on in five patients. Ten patients received concurrent radiation therapy, and 3 autologous hematopoietic stem cell transplantation. Significant toxicities have not been observed. One patient (9.1%) progressed. Two patients had relapse (18.2%), and one patient died (9.1%). Ten patients (90.9%) are alive with a median time follow-up 2.6 years (range 1.2 to 9.2 years). The estimated 2-year event-free survival and overall survival for the whole group were 68.2% and 85.7%, respectively.

**Conclusions:**

Pazopanib was well-tolerated in young patients, even when it was administered with chemotherapy and radiation therapy together. Pazopanib turned out to be effective in patients with primary multi-metastatic Ewing sarcoma and particularly could be considered as an option for them. This regimen deserves further investigation.

## Introduction

Ewing sarcoma (ES) accounts for approximately 40% of all bone malignancies in children and young adults and 3% of soft tissue sarcoma (STS). With advances in multimodal therapy, survival rates for patients with primary localized bone disease approach 70%-75% (1, 2). However, despite the use of different treatment regimens patients with primary multi-metastatic bone ES disease have a dismal outcome (3, 4).

Currently, as the conventional chemotherapy possibilities have been exhausted and the efficiency of the existing treatment procedures are inadequate, more often novel drugs based on alternative mechanisms of action are being sought for. One such drug is pazopanib, which was approved in 2009 for the treatment of advanced/metastatic renal cell carcinoma and STS in adult patients (5–8). Pazopanib is a kinase inhibitor drug that blocks the tumor growth and the ability to create metastases by inhibition of angiogenesis (17). Its moderate toxicity profile and the promising results observed in a few studies on extraosseous ES allow to consider that pazopanib may also be a reasonable treatment option for heavily pretreated patients with bone ES (9–11). Therefore, we aimed to evaluate the efficacy and safety of pazopanib administered in parallel with first-line treatment in young patients with primary multi-metastatic bone ES.

## Materials and methods

### Patients

Eleven patients with primary multi-metastatic bone ES were treated during the period 2016 – 2024 at the Mother and Child Institute (Warsaw, Poland). All patients had histological confirmation of bone ES. Prior to the treatment informed consent was obtained from all patients. In cases when minors were involved the consent was obtained from their legal guardians. Approval for this retrospective study (without control group) was obtained in compliance with the international regulations for protection of human research subjects (Bio-ethical Committee at the Mother and Child Institute in Warsaw).

### Treatment

All patients received standard first-line treatment, including chemotherapy based on VIDE or VDC/IE regimen, followed by surgery and/or radiation therapy. Three patients had high-dose chemotherapy (HDCT) followed by autologous hematopoietic stem cell transplantation (aHSCT) according to the national guidelines (multi-metastatic ES and ≤ 14 years). Pazopanib was administered throughout the entire treatment period with a temporary pause for surgical and aHSCT procedures, and was continued alone as a maintenance therapy. It was administered at a dose of 800 mg once a day in patients older than 15 years and/or weighing more than 50 kg. In younger patients, the dose was calculated in proportion to their body weight and was 200 mg once a day for patients weighing 15 - 20kg, 400mg once a day if the weight was 20 - 30kg, and 600 mg once a day at weight 30 - 50kg. Pazopanib treatment was continued for a minimum of 1 year following the completion of standard therapy, or until disease progression, or unacceptable toxicity. Dose reduction was undertaken in the case of CTCAE v. 4.0 grades 3 and 4. Patients developing side effects such as allergic symptoms, diarrhea, or cytopenia were treated symptomatically according to national guidance. Granulocyte colony-stimulating factor (G-CSF) support (5 mcg/kg) was recommended according to the national guidance or as secondary prophylaxis after an episode of febrile neutropenia in the preceding cycle.

### Assessment of response and toxicity

All patients had standard tumor imaging, including ultrasonography (USG), computed tomography (CT), magnetic resonance imaging (MRI), bone radiography (RTG), or positron emission tomography (PET), as indicated prior to starting chemotherapy and every three courses, always before surgery and radiation therapy. Physical examination and laboratory evaluation were performed prior to each cycle or weekly when indicated. All toxicities were documented from day 1 of pazopanib administration until the end of therapy. WHO criteria were used to evaluate the response. Complete response (CR) was defined as no signs of disease. Partial response (PR) was defined as at least 50% decrease in all measurable lesions (primary or metastases). Progressive disease (PD) was defined as at least 20% increase in the size of any lesions or the development of new lesions. Stable disease (SD) was defined as an absence of CR, PR or PD.

### Statistical methods

Descriptive statistics were used to summarize the characteristics of a data set. Overall survival (OS) was defined as the time interval from the date of diagnosis to the date of death or to the last follow-up date. Event-free survival (EFS) was defined as the time interval from the date of diagnosis to the date of disease progression, recurrence, second malignancy, death or to date of last follow-up for patients without events. Results’ distributions were estimated using the Kaplan-Meier method. Statistical analysis was performed using Python version 3.12.2 in Visual Code Studio version 1.97.0.

## Results

### Patients and treatment

Between 2016 and 2024, 11 patients (8 female, 3 male) with primary multi-metastatic bone ES were referred for treatment to the Mother and Child Institute. The patient clinical and treatment characteristics are shown in **Table 1**. The median age at the time of diagnosis was 13.8 years (range 4.7 to 17.5 years); Eight patients (72.7%) were ≤ 15 years at the time of diagnosis. The most common primary site was the axial location. Eleven (100.0%) patients had metastasis to other bones only, two (18.2%) had bone marrow metastases, and eight (72.7%) had additional lung metastases. All patients received the first-line treatment (VIDE or VDC/IE regimen) parallel with oral pazopanib. Ten (90.9%) patients received concurrent radiation therapy. The primary tumor was operated on in five (45.5%) patients. Five of the five patients (100%) undergoing surgery achieved negative resection margins. Poor histological response was found in one patient (20.0%). Two (25%) patients with lung disease underwent thoracotomy after neoadjuvant chemotherapy, and seven (87.5%) of them subsequently received whole lung radiation therapy. Pazopanib was administered on average 1.7 years (range 0.9 to 5.1).

**Table 1:** Patient characteristics.

Patient characteristics	Feature	N [%]
Gender	Male Female	3 (27.3%) 8 (72.7%)
Median age in years		13.8
Range in years		4.7-17.5
Age	≤15 years >15 years	8 (72.7%) 3 (27,2%)
Primary tumor location	Upper limb Axial Lower limb	1 (9.1%) 7 (63.6%) 3 (27.2%)
Tumor volume	≤200 ml >200 ml	3 (30.0%) 7 (70.0%)
Metastasis location	Lungs Bones Bone marrow Lymph node	8 (72.7%) 11 (100.0%) 2 (18.2%) 1 (9.1%)
Chemotherapy	VIDE IE/VDC	2 (18.2%) 9 (81.8%)
Bone marrow transplant	Yes No	3 (27.3%) 8 (72.3%)
Recurrence	Local Distant Combined	1 (33.3%) 1 (33.3%) 1 (33.3%)
Progression on primary therapy	Yes No	1 (9.1%) 10 (90.9%)

### Toxicity and outcome

Pazopanib was well tolerated. Significant toxicities have not been observed. The most common minor complications included mild to moderate hematologic toxicities (such as transient neutropenia or thrombocytopenia) and gastrointestinal symptoms (including nausea, abdominal discomfort, or diarrhea), which were observed in seven patients. All adverse events were managed symptomatically. Among these patients, the pazopanib dose was reduced by half, and the treatment was continued. There were no other toxicities.

**Figure 1:**
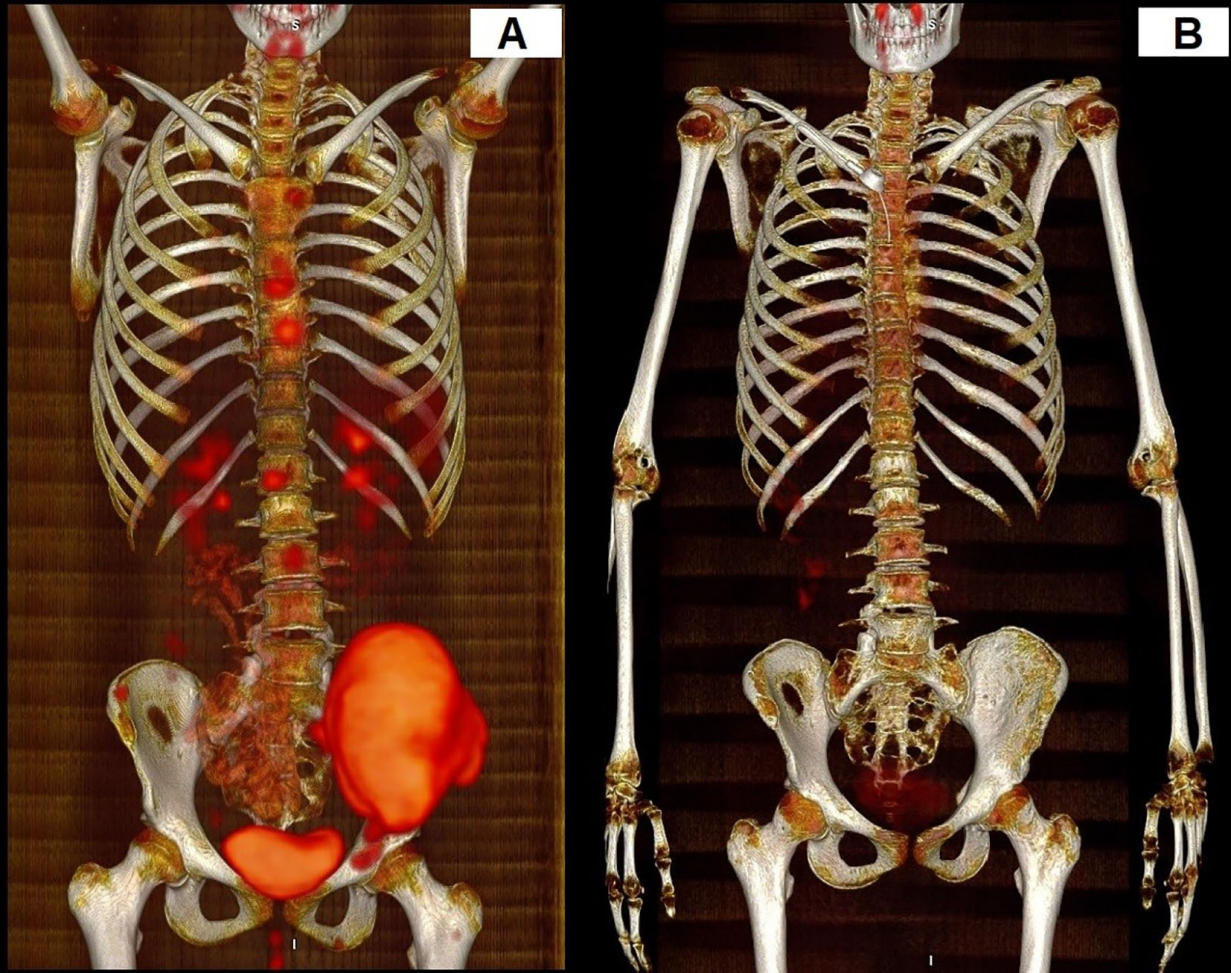
Response to the treatment in PET-CT. The patient with multisystem bone ES. **(A)** PET-CT before the treatment. **(B)** PET-CT after treatment.

**Figure 2:**
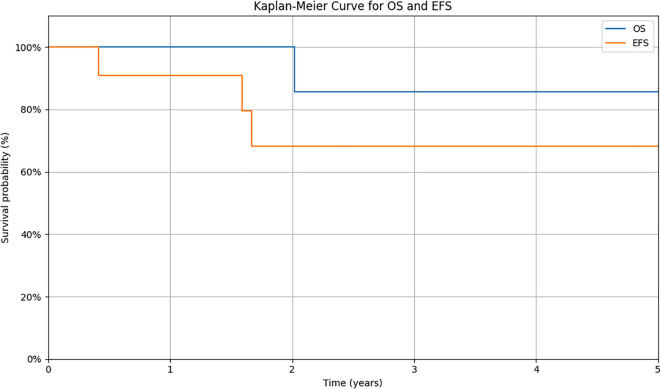
Kaplan-Meier survival curves for overall survival (OS) and event-free survival (EFS) in the studied cohort. Survival probabilities were estimated using the Kaplan-Meier method. The y-axis shows the estimated probability of survival, while the x-axis represents the time from diagnosis (in years). OS is defined as the time from diagnosis to death from any cause or to the last follow-up. EFS is defined as the time from diagnosis to the occurrence of an event (relapse, progression, second malignancy, death, or to the date of last follow-up for patients without events).

Response to the treatment was confirmed in PET-CT in all patients (**Figure 1**). A median time follow-up is 2.6 years (range 1.2 to 9.2 years). At the time of analysis, ten (90.9%) patients were alive. 6 still receiving pazopanib. Progression during the first-line treatment was noted in one (9.1%) patient. Recurrence was detected in two patients (18.2%), and one patient (9.1%) died. Two-year EFS and OS estimates for this group of patients were 68.2% and 85.7%, respectively (**Figure 2**).

## Discussion

Despite the involvement of many people, randomized and non-randomized clinical trials, patients with primary multi-metastatic ES still have a dismal prognosis (1–4). In our previous study, the 5-year OS was estimated at 42% and EFS at 36%., and the 5-year OS for refractory relapsed ES was 23.33% (2, 13). Only patients with isolated lung metastases had a better outcome (5-year EFS 55%), which is consistent with literature data (14). Moreover, the intensification of chemotherapy by using high-dose chemotherapy followed by bone marrow transplantation has not brought the expected results (15). It seems, however, that new strategies may lead to less burdensome treatment with fewer side effects, especially since in many cancers the inclusion of targeted therapies has allowed for more effective and less toxic treatment (16). However, targeted therapies sometimes do not work well enough as monotherapy, so they have been combined with other procedures, such as chemotherapy.

Here, we have presented our results using the combination of standard first-line treatment and pazopanib in the management of primary multi-metastatic bone ES and have confirmed its efficacy. A high response rate (ten patients, 90.9%) was noted in this population, and toxicity was acceptable. Importantly, according to our knowledge, it is one of the first reported studies using pazopanib in this group of patients. Previous studies have described the efficacy of this drug in patients with renal cell sarcoma, STS, and EES [6–11]. Dembla et al. have shown the results of the use of pazopanib combined with other targeted drugs, among others, in bone sarcoma (osteosarcoma, chondrosarcoma, and Ewing sarcoma), but that study group consisted mainly of adult patients, median age was 32.5 years and pazopanib was used primarily in heavily pretreated patients. Our study is the first one to include very young patients. We had seven (63.6%) patients under the age of 15, and the youngest patient on the pazopanib treatment starting day was only 5.1 years old. Previous studies have depicted elderly patients, and the youngest patient treated with pazopanib was a 14-year-old (12). Moreover, in the Dembla study mentioned above, responses were generally short-lived, and treatment was associated with limited disease control. In contrast, in our study, pazopanib was administered earlier in the treatment course and in combination with standard therapy. We observed a higher response rate (90.9%) and better short-term survival outcomes (2-year OS 85.7%), suggesting that younger patients may tolerate pazopanib better and potentially benefit more when it is introduced earlier in the disease course.

Pazopanib is a multi-kinase inhibitor drug approved for the treatment of renal cell carcinoma and non-GIST STS after failure of the first line of therapy. Until recently, it was not taken into consideration when choosing therapy in ES patients. Over the last few years, a few studies have been started to describe the efficacy of pazopanib in patients with ES. Attia and Yamamoto, in two different studies, described a temporary response to pazopanib in men over 60 years old with heavily pre-treated ES (10, 11). In 2018, Mori depicted a complete response to pazopanib in a 17-year-old girl with metastatic extraosseous ES (9). In our study, we noted a high response rate (ten patients, 90.9%). The estimated two-year EFS and OS for our group were 68.2% and 85.7%, respectively, which is higher than our previous report (2).

Unfortunately, a limitation of using tyrosine kinase inhibitors (TKI) in Ewing’s sarcoma is the often lack of identified kinase mutations, which significantly hampers the ability to select patients who might particularly benefit from this therapy. Additionally, some inhibitors cause side effects, which raises concerns about their use, especially in children. What is worth emphasizing, in our study, we observed only minor complications, and no severe side effects were observed, and the average age at which treatment with pazopanib was initiated was 13.2 years.

In our study, in ten patients (90.9%), parallel with pazopanib, radiation therapy was applied. It is known that combining different treatment methods can increase the effectiveness of cancer therapy. Combining kinase inhibitors with radiotherapy may have a radio sensitizing effect, meaning it increases the sensitivity of cancer cells to radiation by impeding DNA repair, blocking cell survival pathways, and inhibiting angiogenesis. Pazopanib, in particular, may enhance the efficacy of both chemotherapy and radiation therapy through multiple mechanisms. As a multi-targeted tyrosine kinase inhibitor that blocks VEGFR, PDGFR, and c-KIT, pazopanib reduces angiogenesis and can transiently normalize the tumor vasculature. This normalization improves intratumoral blood flow, enhances oxygenation, and facilitates more effective delivery of chemotherapeutic agents. Improved oxygenation also increases tumor cell sensitivity to radiation, which depends on oxygen for maximal cytotoxicity. Therefore, pazopanib may not only exert a direct anti-tumor effect but also act synergistically with conventional treatments to increase their efficacy. Given pazopanib’s anti-angiogenic activity, it is conceivable that its use in earlier disease stages could help suppress the formation of new metastatic sites. By inhibiting neovascularization, pazopanib may impair the establishment of pre-metastatic niches and the progression of micrometastatic disease (17).

Moreover, the potential for combining pazopanib with immunotherapy, such as immune checkpoint inhibitors, is an emerging area of interest. Anti-angiogenic agents like pazopanib have been shown to modulate the tumor immune microenvironment by normalizing vasculature, reducing immunosuppressive cells such as Tregs and MDSCs, and enhancing CD8+ T-cell infiltration in several cancer types. Although such combinations have been explored in sarcomas and renal cell carcinoma, they have not yet been studied in Ewing sarcoma. Nevertheless, our findings, together with the known immunomodulatory effects of pazopanib, provide a rationale for future investigation of such combinatorial approaches in high-risk ES (18–20).

Combining pazopanib with other targeted agents, such as IGF - 1R inhibitors, represents a promising but still experimental approach in the treatment of Ewing sarcoma. IGF - 1R signaling is a known driver in ES pathogenesis, and its inhibition has shown some clinical activity. However, responses to IGF - 1R inhibitors alone have been modest and often transient. Given that pazopanib targets angiogenesis and tumor vasculature, while IGF - 1R inhibitors affect proliferative signaling, the combination may offer complementary therapeutic effects. Early clinical attempts to combine pazopanib with other targeted therapies in sarcoma patients, including those with ES, have shown limited success so far and require careful evaluation of toxicity and pharmacologic interactions (21, 22).

However, combining treatment methods can also lead to increased toxicity, which may affect the timing and implementation of standard therapeutic procedures (23, 24). In our group, we did not observe significant complications from such a combination. Currently, clinical trials are underway investigating the combination of kinase inhibitors with radiotherapy in various cancers (mainly gliomas, lung, head and neck cancers, and sarcomas) (25–27). In Ewing’s sarcoma, these are still mainly preclinical studies, but the results are promising.

The outcome of primary metastatic ES is dismal, highlighting the importance of developing new treatment strategies in multi-metastatic ES. The use of targeted therapy in pediatric ES is a rapidly developing area of research that offers hope for more effective and less toxic treatments. Combination therapy (kinase inhibitors and conventional treatment) appears to be the most promising approach. Predictive biomarkers are needed to tailor targeted therapy to the specific molecular profile of the tumor. Personalized medicine (e.g., RNA or proteome analysis of the tumor) may help identify when kinase inhibitors are most effective (28). Due to the rarity of the disease in children, large randomized clinical trials are lacking. Numerous preclinical and clinical studies are currently underway, which we hope will lead to the development of more effective treatment regimens in the future. Our results suggest that while awaiting a breakthrough, it is possible to improve prognosis in patients with metastatic ES by utilizing currently available strategies.

We realize that our study is limited and can be biased because of the small group and retrospective data. Nevertheless, the study confirms that pazopanib is well-tolerated in young patients with ES. It could be safely used in parallel with chemotherapy and radiation therapy. This schedule seems to have the potential to improve outcomes and can be an option for primary metastatic ES patients and should be considered for earlier integration into the treatment algorithm. Further prospective studies are needed to better define the use of pazopanib in the upfront management of these groups of patients.

## Data Availability

The raw data supporting the conclusions of this article will be made available by the authors, without undue reservation.
